# Severe Maternal Stress Exposure Due to Bereavement before, during and after Pregnancy and Risk of Overweight and Obesity in Young Adult Men: A Danish National Cohort Study

**DOI:** 10.1371/journal.pone.0097490

**Published:** 2014-05-14

**Authors:** Lena Hohwü, Jiong Li, Jørn Olsen, Thorkild I. A. Sørensen, Carsten Obel

**Affiliations:** 1 Section for General Practice, Department of Public Health, Aarhus University, Aarhus, Denmark; 2 Section for Epidemiology, Department of Public Health, Aarhus University, Aarhus, Denmark; 3 Institute of Preventive Medicine, Bispebjerg and Frederiksbjerg Hospitals, Copenhagen, Denmark; 4 Novo Nordisk Foundation Center for Basic Metabolic Research, Faculty of Health and Medical Sciences, University of Copenhagen, Copenhagen, Denmark; University of Barcelona, Faculty of Biology, Spain

## Abstract

**Background:**

Perinatal stress may programme overweight and obesity. We examined whether maternal pre- and post-natal bereavement was associated with overweight and obesity in young men.

**Methods:**

A cohort study was conducted including 119,908 men born from 1976 to 1993 and examined for military service between 2006 and 2011. Among them, 4,813 conscripts were born to mothers bereaved by death of a close relative from 12 months preconception to birth of the child (exposed group). Median body mass index (BMI) and prevalence of overweight and obesity were estimated. Odds ratio of overweight (BMI≥25 kg/m^2^) and obesity (BMI≥30 kg/m^2^) were estimated by logistic regression analysis adjusted for maternal educational level.

**Results:**

Median BMI was similar in the exposed and the unexposed group but the prevalence of overweight (33.3% versus 30.4%, p = 0.02) and obesity (9.8% versus 8.5%, p = 0.06) was higher in the exposed group. Conscripts exposed 6 to 0 months before conception and during pregnancy had a higher risk of overweight (odds ratio 1.15, 95% confidence interval (CI): 1.03; 1.27 and odds ratio 1.13, 95% CI: 1.03; 1.25, respectively). Conscripts born to mothers who experienced death of the child’s biological father before child birth had a two-fold risk of obesity (odds ratio 2.00, 95% CI: 0.93; 4.31). There was no elevated risk in those who experienced maternal bereavement postnatally.

**Conclusion:**

Maternal bereavement during the prenatal period was associated with increased risk of overweight or obesity in a group of young male conscripts, and this may possibly be reflected to severe stress exposure early in life. However, not all associations were clear, and further studies are warranted.

## Introduction

In middle to high income parts of the world, the prevalence of obesity has increased for unknown reasons and in the last decades to epidemic levels in many countries [1]. Exposure to stress during fetal life may program susceptibility to obesity in an obesogenic environment [2], [3] and may be one of the many possible explanations for this epidemic. A likely mechanism may be that maternal stress-induced excess of the hormone cortisol pass the placental barrier early in pregnancy and program the fetal hypothalamic-pituitary-adrenal axis [4], [5]. Measuring stress in large scale studies is a major methodological challenge, especially if considering coping capacity [6], but severe stressors like bereavement is well described. Bereavement is classified as the most stressful, but rare, life event [7] and it will cause severe stress in almost all exposed, regardless of coping strategies [8], and especially if caused by loss of a child or spouse [9].

We have previously demonstrated an association between prenatal bereavement and overweight in school children from 12 to 15 years [10]. This may indicate a programming effect of prenatal stress on obesity triggered by puberty and calls for an examination of the association following the exposed to adulthood.

If the association is causal one would expect the sensitivity to perinatal stress exposure to vary across different time windows and the level of exposure to the relationship to the deceased [11]. We examined whether maternal bereavement during pregnancy and in expanded pre- and post-natal time windows was associated with overweight and obesity in young men attending a routine examination for military service.

## Methods

### Study Population and Data Collection

We conducted a cohort study among 119,908 Danish men born in 1976–1993 and examined at conscription in 2006–2011. All men in Denmark are liable for military service and are called for a conscript board examination around the age of 18 years. Conscripts may request a later appointment. Conscripts may be exempt for examination if they provide medical documentation for conditions that would disqualify them for military service (16%) [12]. The examination includes standard measurement of height and weight and all data are registered in the conscript file [13]. All live born children and new residents in Denmark are assigned a unique civil personal registration number, allowing accurate linkage of data between registries [14]. This makes it possible to track individual data concerning demography, vital status, family relations, social and economic conditions, and health [14]. We linked conscript files with the Danish Civil Registration System [15], Danish Medical Birth Register [16], the Danish National Patient Register [17], and the Integrated Database for Longitudinal Labour Market Research database [18]. We identified a study population of 119,908 males aged 17 to 31 years presenting at the conscript board health examination. Data on the 119,908 conscripts was linked to their parents, siblings, grandparents, and mother’s siblings in whom information on the date of death could be retrieved. Those exposed to bereavement were found by identifying children born to mothers who experienced death of a close relative (partner, child, sibling, or parent) from 12 months before the estimated time of conception to the birth of the index child (death of a partner before conception not included). A priori we decided the time window to be from one year before conception until birth due to potential allostatic load. A total of 4,813 conscripts were included in the exposed group, of these 1,859 were exposed during pregnancy. The remaining 115,095 conscripts constituted the unexposed cohort.

The conscript file information on height (over 1.4 meters and below 2.5 meters) measured without shoes and weight (over 30 kg and under 250 kg) measured in underwear was used to calculate the outcome variable body mass index (BMI), defining overweight as BMI≥25 kg/m^2^ and obesity as BMI≥30 kg/m^2^ in accordance with the World Health Organisation [1].

Information on potential confounders was collected from the Danish Medical Birth Register on: parity (1, 2, >2) and gestational age (<37 weeks, ≥37 weeks, unknown). Information on maternal age (<27 years, 27–30 years, >30 years), and cohabitating status (yes, no) at birth was retrieved from the Danish Civil Registration System whereas information of maternal socio-economic factors at time of birth was obtained from the Integrated Database of Longitudinal Labour Market Research database: education (≤9 years, 10–11 years, ≥12 years) and income (0, first, second, and third tertile). Birth weight was considered a potential intermediate factor rather than a potential confounder.

### Statistical Analysis

The baseline characteristics between exposed and unexposed was tested using chi-square test.

Median BMI with corresponding interquartile ranges and prevalence of overweight and obesity with corresponding 95% confidence intervals (CI) and P values were estimated. Multiple logistic regression models were used to estimate the odds ratio for overweight and obesity with corresponding 95% CI. The 10% change-in-estimate was used for confounder-selection to be controlled in the models [19]. We performed stratified analyses into five sub-groups according to timing of the exposure, categorizing the time period from 12 months preconception through childbirth to 24 months after birth: 12 to 7 months preconception, 6 to 0 months preconception, during pregnancy, 0 to12 months after birth, and 13 to 24 months after birth. Death of the biological father was categorized into the three latter categories. Death of a close relative during pregnancy was further categorized to the time periods 1^st^, 2^nd^ or 3^rd^ trimester. The overall estimate, death of a close relative, was categorized into three groups, according to the relation to the deceased: death of biological father, death of a child, and the death of other close relatives (mother’s siblings and parents). Stratified analyses for trimester and the mother’s relation to the deceased close relative during pregnancy are presented in Table S1, but due to a limited number of events, it was only possible to estimate unadjusted odds ratio for overweight and obesity. As bereavement was distributed evenly over the years no stratification by years are reported. A *P* value of <0.05 was considered statistically significant.

The Danish Data Protection Agency approved the study (J.nr. 2008-41-2555, J.nr.2008-41-2680). In Denmark, ethical approval is not required for de-identified register-based research.

Data analyses were conducted in Stata version 11 (StataCorp LP, College Station, TX, USA).

## Results

Table 1 presents baseline characteristics at birth. The median age of the conscripts was19 years. The prevalence of mothers with three or more births was higher in the exposed (20.5%) than the unexposed (14.0%) (p<0.01). The prevalence of mothers with a low educational level was 37.8% for exposed conscripts and 33.7% for unexposed conscripts (p<0.01).

**Table 1:** Baseline characteristics of the study population at birth (N = 119,908).

	Exposed		Unexposed		
	(n = 4,813)		(n = 115,095)		
	n	(%)	n	(%)	*P* value^a^
**Gestational age, week**					
<37	256	(5.4)	5,743	(5.1)	
≥37	4,483	(94.6)	106,812	(94.9)	0.36
**Maternal age, years**					
≤26	1,581	(32.8)	42,863	(37.2)	
27–30	1,684	(35.0)	40,024	(34.8)	
>30	1,548	(32.2)	32,206	(28.0)	<0.01
**Birth weight, gram**					
<2,500	219	(4.6)	4,780	(4.2)	
2,500–3,249	1,149	(24.2)	27,681	(24.5)	
3,250–4,000	2,490	(52.3)	58,574	(51.9)	
>4,000	900	(18.9)	21,952	(19.4)	0.47
**Parity**					
1	1,845	(38.3)	54,430	(47.3)	
2	1,981	(41.2)	44,521	(38.7)	
≥3	987	(20.5)	16,125	(14.0)	<0.01
**Maternal education, years**					
Primary (≤9)	1,820	(37.8)	38,756	(33.7)	
Secondary (10–11)	1,542	(32.1)	38,880	(33.8)	
High (≥12)	1,362	(28.3)	34,140	(29.7)	
Unknown	86	(1.8)	3,231	(2.8)	<0.01
**Maternal income**					
≤0	100	(2.1)	2,299	(2.0)	
Low (⅓)	719	(14.9)	17,062	(14.9)	
Middle (⅓)	1,983	(41.2)	45,813	(39.8)	
High (⅓)	1,970	(41.0)	47,971	(41.7)	
Unknown	38	(0.8)	1,862	(1.6)	<0.01
**Maternal cohabition status**					
Cohabitation	2,411	(50.1)	58,443	(50.8)	
Single	2,361	(49.1)	54,702	(47.6)	
Unknown	38	(0.8)	1,862	(1.6)	<0.01

Exposed: Children born to mothers who experienced the death of a close relative during the time period from 12 months preconception through birth.

Unexposed: Children born of mothers who did not experience the death of a close relative during the time period from 12 months preconception through birth Close relative: partner, child, mother’s sibling, or mother’s parents.

a Chi-square test.

The median BMI and prevalence of overweight and obesity are shown in Table 2. The median BMI was the same in children born to mothers bereaved by death of a close relative during pregnancy (23.2 kg/m^2^) and unexposed children (23.1 kg/m^2^). The overall prevalence of overweight was statistically significantly higher in children born to mothers bereaved by death of a close relative during pregnancy than unexposed children (33.3% (95% CI: 30.9%; 35.2%) versus 30.4% (95% CI: 30.2%; 30.7%), p = 0.02). The overall prevalence of obesity during pregnancy was higher, but not statistically significant, in exposed than unexposed (9.8% (95% CI: 8.4%; 11.1%) versus 8.5 (95% CI: 8.3; 8.7), p = 0.06). In children born to mothers bereaved by death of her sibling or parent during pregnancy, the prevalence of overweight was statistically significantly higher (32.8%, p = 0.04) than the unexposed (30.5%) whereas the prevalence of obesity was not. For conscripts born by mothers who experienced death of the biological father during pregnancy, the prevalence of overweight and obesity was increased by 11.7% (p = 0.09) and 9.3% (p = 0.03), respectively compared with the unexposed. Contrary, if the mother lost a child the prevalence of overweight and obesity was statistically significantly higher in conscripts exposed 6 to 0 month before conception (Table 2).

**Table 2:** The median body mass index, and prevalence of overweight and obesity of exposed and unexposed children, according to timing of bereavement and the mother’s relation to the deceased close relative (N = 119,908).

	Body Mass Index								
				≥25 kg/m^2^			≥30 kg/m		
Maternal bereavement	n	median	(iqr)	%	(95% CI)	*P* a	%	(95% CI)	*P* a
**Any close relative**									
Unexposed	109,702	23.1	(4.8)	30.4	(30.2; 30.7)		8.5	(8.3; 8.7)	
12 to 7 months preconception	1,415	23.0	(4.6)	29.3	(26.9; 31.6)	0.34	9.5	(8.0; 11.1)	0.18
6 to 0 months preconception	1,541	23.2	(4.8)	33.6	(31.2; 36.0)	0.01	9.3	(7.8; 10.7)	0.28
Pregnancy	1,859	23.2	(4.7)	33.3	(30.9; 35.2)	0.02	9.8	(8.4; 11.1)	0.06
0 to 12 months postnatal	2,602	23.5	(4.7)	29.6	(27.9; 31.4)	0.01	8.8	(7.7; 9.9)	0.58
13 to 24 months postnatal	2,789	23.0	(4.6)	30.9	(29.5; 32.6)	0.62	8.5	(7.5; 9.5)	0.99
**Trimester, any** **close relative**									
1^st^ trimester	623	23.1	(5.2)	33.2	(30.2; 30.7)	0.13	10.4	(8.0; 12.8)	0.09
2^nd^ trimester	670	23.2	(4.7)	32.4	(28.9; 35.9)	0.28	9.4	(7.2; 11.6)	0.42
3^rd^ trimester	590	23.4	(4.8)	33.7	(29.9; 37.5)	0.09	9.3	(7.0; 11.7)	0.49
**Sibling or parents**									
Unexposed	110,857	23.1	(4.7)	30.5	(30.2; 30.7)		8.5	(8.4; 8.7)	
12 to 7 monthspreconception	1,140	23.0	(4.6)	28.5	(25.9; 31.2)	0.16	8.8	(7.2; 10.5)	0.71
6 to 0 monthspreconception	1,170	22.9	(4.4)	32.7	(30.0; 35.4)	0.10	8.3	(6.7; 9.9)	0.77
Pregnancy	1,763	23.1	(4.6)	32.8	(30.6; 35.0)	0.04	9.6	(8.2; 11.0)	0.11
0 to 12 months postnatal	2,418	23.2	(4.9)	29.5	(27.7; 31.3)	0.30	8.8	(7.6; 9.9)	0.67
13 to 24 months postnatal	2,560	23.0	(4.6)	30.9	(29.1; 32.7)	0.61	8.5	(7.4; 9.6)	0.94
**Child**									
Unexposed	118,986	23.1	(4.4)	30.5	(30.2; 30.7)		8.5	(8.4; 8.7)	
12 to 7 months preconception	281	23.1	(5.5)	32.0	(26.8; 37.8)	0.52	12.9	(9.0; 16.8)	0.01
6 to 0 monthspreconception	378	23.6	(5.7)	36.5	(31.5; 41.2)	0.01	11.9	(8.7; 15.2)	0.02
Pregnancy	57	23.0	(5.0)	33.3	(21.0; 45.7)	0.64	5.3	(−0.6; 11.1)	0.38
0 to 12 months postnatal	93	23.5	(4.9)	32.3	(22.7; 41.8)	0.71	8.6	(2.9; 14.3)	0.98
13 to 24 months postnatal	113	23.3	(4.4)	31.0	(22.4; 39.5)	0.90	8.9	(3.6; 14.1)	0.90
**Partner**									
Unexposed	119,618	23.1	(4.7)	30.5	(30.2; 30.7)		8.5	(8.4; 8.7)	
Pregnancy	45	23.7	(7.7)	42.2	(27.6; 56.8)	0.09	17.8	(6.5; 29.1)	0.03
0 to 12 months postnatal	111	22.7	(5.1)	28.8	(20.4; 37.3)	0.71	9.0	(3.7; 14.4)	0.86
13 to 24 months postnatal	135	23.3	(4.6)	28.4	(20.7; 36.0)	0.60	7.5	(3.0; 11.9)	0.66

Close relative: partner, child, mother’s sibling, or mother’s parents iqr: interquatile range CI denotes confidence interval.

a *P* value. Chi-square test.

**Figure 1:**
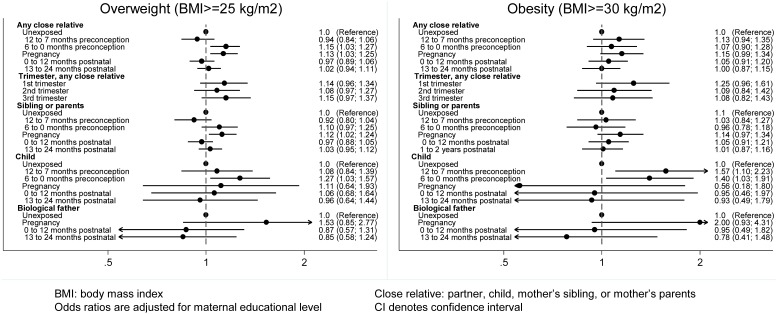
Adjusted odds ratio (95% CI) for overweight and obesity in young male conscripts exposed to maternal bereavement (N = 119,908).

Maternal level of education was the only identified potential confounder that induced a change-of-estimate of more than a 10% difference in the crude and adjusted estimate and was therefore included in the multiple logistic regression models. Overall, conscripts born to mothers experiencing death of a close relative had a 15% statistically significantly higher risk of being overweight (odds ratio 1.15, 95% CI: 1.03; 1.27) if exposed 6 to 0 months before conception, and a 13% statistically significantly higher risk (odds ratio 1.13, 95% CI: 1.03; 1.25) if exposed during pregnancy (Figure 1). The overall increased risk of obesity was for conscripts born by mothers bereaved 12 to 7 months before conception (odds ratio 1.13, 95% CI: 0.94; 1.35) and during pregnancy (odds ratio 1.15, 95% CI: 0.99; 1.34). The strongest, though not statistically significant, association for overweight and obesity was seen for loss of the biological father during pregnancy (odds ratio 1.53, 95% CI: 0.85; 2.77 and odds ratio 2.00, 95% CI: 0.93; 4.31, respectively) (Figure 1). The loss of a child 6 to 0 months before conception was associated with a statistically significantly increased risk of overweight; This association was even higher for obesity (Figure 1). If the mother lost a child during pregnancy the risk of overweight was not significantly increased (odds ratio 1.11, 95% CI: 0.64; 1.93), whereas the risk for obesity was low, but with wide confidence intervals (odds ratio 0.56, 95% CI: 0.18; 1.80) (Figure 1).

Children born to mothers experiencing death of a close relative postnatally did not have an increased risk of overweight and obesity (Figure 1).

Conscripts exposed during fetal life in the 1^st^ and 3^rd^ trimester had the highest risks of overweight. The highest risk for obesity was observed in those exposed in the 1^st^ trimester (odds ratio 1.25, 95% CI: 0.96; 1.61) (Figure 1). We found an increased, though not statistically significant, risk for obesity for those exposed during the 1^st^ trimester (odds ratio 1.28, 95% CI: 0.39; 4.26) among mothers losing a child during pregnancy (Table S1).

## Discussion

Children born to mothers bereaved during pregnancy had an overall higher risk of overweight in early adult life than unexposed children; They also had an increased risk of overweight if born to mothers experiencing loss of a close relative 6 to 0 months before conception. As expected, we found the strongest point estimate when bereaved by loss of a biological father during pregnancy, whereas the results of loss of a child was less clear. Conscripts exposed during fetal life in the 1^st^ trimester had an increased risk, but not statistically significantly, of both overweight and obesity. The results for maternal bereavement in the first two years postnatally showed no substantially increased risk of overweight and obesity in the conscripts. Our findings may contribute to the understanding of the association between exposures to prenatal stress by maternal bereavement from 12 months before conception through child birth and the risk of overweight and obesity in the offspring.

The results were in line with our previous findings in school children [10] where we observed increased risk of overweight from the age of 12 years (odds ratio 1.68, 95% CI: 1.08; 2.61). A cross-sectional study of serious psychological distress affecting mothers early in life found a higher risk for obesity in 3-year-old children [20]. An even stronger association was observed in another cross-sectional study of family stress and maternal, but not paternal, attachment style and overweight in 3-year-old Swedish children [21], but these designs provided no direction of the association. Bereavement during the first six years of life did not seem to have any influence on overweight in 7–13-year-old schoolchildren [22], but the long-term effect of severe stress in childhood remains unknown and should be investigated in cohorts with prolonged follow-up.

Previous findings of the association between more common sources of maternal stress early in life and risk of overweight in the offspring found that maternal distress as a combined measure of anxiety, depression, and stress during pregnancy was not significantly associated with overweight in offspring at the age of 7 years (odds ratio 1.06, 95% CI: 0.96; 1.18) [23]. Another study of maternal distress 6 months postpartum also found no association with overweight in offspring at the age of 7 years [24]. These findings may be due to less severe stress-exposures than bereavement and that the follow-up time was too short to demonstrate an association later in childhood.

We used maternal bereavement to obtain a large exposure contrast concerning stress. Furthermore, the longitudinal design, the well-defined measurement of exposure, the unbiased measurement of outcome, and independently collected exposure and confounder data were strengths of our study. However, the study also has limitations. First, we have estimated the association only in men, and the findings may not be extrapolated to women. Secondly, the study only included those who survived until the age of conscription, which may cause a bias towards the null, but the bias is small given the low morbidity in this age group. Third of all, if some obese men were exempted from the conscription examination, we may have underestimated the association if this should be associated with prenatal exposure which we find unlikely. Finally, the stress exposure elicited by bereavement may be a bit unclear and we cannot rule out other factors that may have caused stress. Further, the exposure was composed of expected and sudden cause of death. The death of a close relative after a prolonged and possibly painful disease may for some be followed by relief, whereas sudden and unexpected death may generate a different stress exposure. If sudden death induces a higher stress exposure, we may have underestimated the association for this exposure. Stratified analyses of unexpected causes of death alone were not possible because of few such events. Moreover, the association between maternal bereavement and obesity in offspring could be due to confounding by familial predisposition to overweight and obesity. The most common causes of death of the biological father in this study were fractures (36%), mainly due to accidents, cancer (11%), and myocardial infarction (9%): The latter may be related to obesity [25]. As no detailed health information on the biological father was available, we had no possibility to evaluate confounding by familial predisposition to overweight and obesity. We also had limited opportunies to address if time-varying confounders, and especially changes in family dynamics released to loss of a father, was of concern and could in part explain the association.

The increased risk for overweight and obesity in those exposed during pregnancy indicate that pregnancy is a sensitive window for susceptibility to obesity. During normal conditions the placenta enzyme 11β-hydroxysteroid dehydrogenase type 2 acts as a fetal-placental barrier preventing the majority (80–90%) of the maternal glucocorticoid, cortisol, from crossing the placental barrier in an active form [26], [27]. However, the concentration of cortisol in the fetus is correlated to maternal level of cortisol during pregnancy [28], and studies in human [29] and rats [30], [31] have shown that maternal prenatal stress can lead to down-regulation of the 11β-hydroxysteroid dehydrogenase type 2. As a result, the concentration of cortisol in the fetus may influence the fetal development of hypothalamic-pituitary-adrenal axis [4], [5]. Animal studies in antenatal maternal nutrient-restricted sheep [32] and stress-responded maternal stress-sensitive mice [11] showed an association with increased body weight in the offspring. This could be explained by transcriptomic and epigenetic changes in the hypothalamus, a portion of the brain involving the regulation of appetite and food intake [33]. These changes are related to increased susceptibility to overweight and obesity [33], and consequently altered programming effect of growth in the offspring.

The association between maternal bereavement and overweight from the age of 12 years [10] and in young adulthood may indicate that the long-term programming is modified by puberty development [34]. Prenatal stress may affect the timing or the velocity of pubertal growth spurt [35] and thereby the risk of future overweight or obesity [36]. In addition, a lifestyle in adolescents characterized by sedentary behavior, unhealthy eating patterns [37], and lack of sleep may contribute to an increased BMI. Also hormonal interaction during sexual maturation could increase the risk of overweight or obesity at puberty [38], and our study suggests that obesity could extend further to later life, including early adulthood.

Although timing of death (bereavement) is well recorded, it might often be different from the timing of stress exposure. It is not well documented how long bereavement lasts and substantial individual variation is expected. Most deaths occur after a shorter or longer time period of severe illness and pain also affecting close relatives and friends. The perception of stress is influenced by the individual experiences, genetics and behavior, and the long-term effect of the psychological response to stress can lead to an allostatic load that might explain the increased risk of overweight and particularly obesity in conscripts born to mothers who lost a child 12 to 0 months before conception [39]. We found consistent associations between bereavement and overweigth as well as obesity except for an insignificant negative association between loss of a child during pregnancy and obesity. The negative association may be due to coincidence and was not found in our previous study. It can be speculated that the loss of another child may elicit an extended protective lifestyle that may have a deprogramming effect. This needs further investigation.

In conclusion, our overall results suggest that severe prenatal stress, both preconceptional and gestational, related to maternal bereavement is associated with increased risk of overweight in a group of young male conscripts. Bereavement due to loss of a child before conception was associated with increased risk of overweight and obesity. Our findings may reflect that these associations could be due to fetal programming perhaps induced by long-lasting epigenetic modifications. However, we found that not all associations were clear when stratifying for the mother’s relation to the deceased close relative and further studies are warranted.

## Supporting Information

Table S1 **Unadjusted odds ratio for overweight and obesity in children exposed to maternal bereavement during pregnancy, stratified for trimester and the mother’s relation to the deceased close relative.**
(DOCX)
