# Supplementary material for: Severe Maternal Stress Exposure Due to Bereavement before, during and after Pregnancy and Risk of Overweight and Obesity in Young Adult Men: A Danish National Cohort Study
Source: PLoS One. 2014 May 14;9(5):e97490. doi: 10.1371/journal.pone.0097490 (PMC4020839; doi:10.1371/journal.pone.0097490)
Supplement: Table S1 — Unadjusted odds ratio for overweight and obesity in children exposed to maternal bereavement during pregnancy, stratified for trimester and the mother’s relation to the deceased close relative. (DOCX) [file pone.0097490.s001.docx]

**Table S1**. Unadjusted odds ratio for overweight and obesity in children exposed to maternal bereavement during pregnancy, stratified for trimester and the mother’s relation to the deceased close relative.

|  |  | BMI ≥25 kg/m^2^ | |  | | BMI ≥30 kg/m^2^ | |
| --- | --- | --- | --- | --- | --- | --- | --- |
| Maternal bereavement | n | Odds ratio | (95% CI) | |  | Odds ratio | (95% CI) |
| **Sibling or parents** |  |  |  | |  |  |  |
| Unexposed | 118,131 | 1.0 | (Reference) | | | 1.0 | (Reference) |
| 1^st^ trimester | 583 | 1.15 | (0.97; 1.36) | | | 1.21 | (0.92; 1.58) |
| 2^nd^ trimester | 641 | 1.10 | (0.94; 1.30) | | | 1.11 | (0.85; 1.45) |
| 3^rd^ trimester | 553 | 1.12 | (0.94; 1.34) | | | 1.14 | (0.86; 1.51) |
| **Child** |  |  |  | |  |  |  |
| Unexposed | 119,846 | 1.0 | (Reference) | | | 1.0 | (Reference) |
| 1^st^ trimester | 28 | 0.91 | (0.40; 2.07) | | | 1.28 | (0.39; 4.26) |
| 2^nd^ trimester | 15 | 0.57 | (0.16; 2.02) | | | none |  |
| 3^rd^ trimester | 19 | 1.66 | (0.67; 4.12) | | | none |  |
| **Biological father** |  |  |  | |  |  |  |
| Unexposed | 119,863 | 1.0 | (Reference) | | | 1.0 | (Reference) |
| 1^st^ trimester | 13 | 1.42 | (0.47; 4.36) | | | 3.21 | (0.88; 11.68) |
| 2^nd^ trimester | 14 | 1.27 | (0.42; 3.78) | | | 2.92 | (0.81; 10.47) |
| 3^rd^ trimester | 18 | 2.28 | (0.90; 5.36) | | | 1.34 | (0.31; 5.82) |

CI denotes confidence interval BMI: body mass index
